# TwiMed: Twitter and PubMed Comparable Corpus of Drugs, Diseases, Symptoms, and Their Relations

**DOI:** 10.2196/publichealth.6396

**Published:** 2017-05-03

**Authors:** Nestor Alvaro, Yusuke Miyao, Nigel Collier

**Affiliations:** ^1^ National Institute of Informatics Department of Informatics Tokyo Japan; ^2^ The Graduate University for Advanced Studies (SOKENDAI) Kanagawa Japan; ^3^ Faculty of Modern & Medieval Languages Department of Theoretical and Applied Linguistics University of Cambridge Cambridge United Kingdom

**Keywords:** Twitter, PubMed, corpus, pharmacovigilance, natural language processing, text mining, annotation

## Abstract

**Background:**

Work on pharmacovigilance systems using texts from PubMed and Twitter typically target at different elements and use different annotation guidelines resulting in a scenario where there is no comparable set of documents from both Twitter and PubMed annotated in the same manner.

**Objective:**

This study aimed to provide a comparable corpus of texts from PubMed and Twitter that can be used to study drug reports from these two sources of information, allowing researchers in the area of pharmacovigilance using natural language processing (NLP) to perform experiments to better understand the similarities and differences between drug reports in Twitter and PubMed.

**Methods:**

We produced a corpus comprising 1000 tweets and 1000 PubMed sentences selected using the same strategy and annotated at entity level by the same experts (pharmacists) using the same set of guidelines.

**Results:**

The resulting corpus, annotated by two pharmacists, comprises semantically correct annotations for a set of drugs, diseases, and symptoms. This corpus contains the annotations for 3144 entities, 2749 relations, and 5003 attributes.

**Conclusions:**

We present a corpus that is unique in its characteristics as this is the first corpus for pharmacovigilance curated from Twitter messages and PubMed sentences using the same data selection and annotation strategies. We believe this corpus will be of particular interest for researchers willing to compare results from pharmacovigilance systems (eg, classifiers and named entity recognition systems) when using data from Twitter and from PubMed. We hope that given the comprehensive set of drug names and the annotated entities and relations, this corpus becomes a standard resource to compare results from different pharmacovigilance studies in the area of NLP.

## Introduction

Corpora annotated for adverse drug events are becoming important in order to train computers to automatically build adverse drug reaction profiles for post marketing surveillance.

Researchers are typically interested in understanding the accuracy of their systems [1-8], whereas at the same time only a limited number of corpora exist [9-12].

Pharmacovigilance (drug safety) systems using texts obtained from the scientific literature have received attention for many years [1,6-8] and since recently researchers started exploring Twitter and other nonscientific texts where patients describe diseases and symptoms [2-4]. However, there is currently no way to systematically compare systems performance across text types.

In this paper we provide a benchmark corpus composed of semantically correct annotations that can be used in natural language processing (NLP) studies and show our approach to produce a comparable corpora using texts from Twitter and PubMed, explaining our strategy for controlling external variables that may affect the sample.

Social media texts are known for containing a high proportion of ungrammatical constructions out of vocabulary words, abbreviations, and metaphoric usage [13], whereas scientific texts are known for the use of specialized vocabulary and well-formed sentences. Secondary key factors involved in a direct comparison are the data selection methods and the topicality [14].

Existing corpora from PubMed and Twitter cannot be directly compared and the goal of this research is to produce a comparable corpus of drug-related sentences targeting at the same set of drugs.

To date, most of the curated corpora for pharmacovigilance come from scientific formal texts obtained from PubMed [15,16], although datasets curated from other scientific resources, such as the Khresmoi project [17], are also available [18].

Since a few years ago, corpora obtained from social media texts started emerging. At first, researchers focused on blogs and forums [19,20] and then on Twitter’s data [3,21,22] due to the high volume of the information it provides, with 310 million monthly active users [23] generating over 500 million tweets per day [24] and also motivated by its “realtime” information, allowing health researchers to potentially investigate and identify new adverse drug event (ADE) types faster than traditional methods such as physician reports.

Researchers have assessed the number of scientific works in PubMed where Twitter data was used [25], finding that the interest in Twitter is growing within the medical domain. Pharmacovigilance sees Twitter as a useful resource in different areas. Messages found in Twitter tweets can help researchers to understand temporal patterns on the drugs usage [26], can provide a good resource for obtaining first-hand experience reports on the drugs use [21], and can be useful in the early detection of prescription medication abuse [27] and adverse events [3,28].

Recent examples where researchers used Twitter to build different corpora are: (1) the corpus built to understand patient experiences at health care facilities [29], (2) the corpus built to measure the public interest and concerns about different diseases [30], and (3) the corpus used to assess the positive or negative attitude toward specific treatments [31]. Although texts written in English have been used very frequently in NLP for pharmacovigilance, texts in Spanish extracted from social media [32] and French clinical texts [33] have been also used.

The work on Twitter and PubMed is an ongoing effort resulting in promising NLP studies on the automatic recognition of medications and adverse events [34,35] and also on the medical question answering [36,37]. By releasing our corpus, we hope other researchers can benefit from it and continue advancing in this area.

## Methods

### Data Selection

For our study, we selected a set of 30 different drugs used in other pharmacovigilance studies [3,5,21,38,39]. Including these drugs allowed us to cover different interests in the research community and also allowed us to account for drugs used to treat very different conditions such as cancer [3], attention deficit disorders [5,21,39], schizophrenia [3,38], or depression [5,21,38,39].

We employed Twitter’s application programming interface (API) to download messages mentioning any of those drug names or their synonyms by running our script from September 7, 2015 to October 10, 2015, obtaining 165,489 tweets. In the case of PubMed, we obtained the list of articles about those drugs by using EuropePMC RESTful Web Services [40], issuing our query on October 21, 2015 to search for texts containing the same keywords that we used when collecting tweets. Once we had the list of PubMed articles, we processed them to extract the sentences containing the drug mentions obtaining 29,435 sentences.

From these sentences, we removed all non-ASCII (American standard code for information interchange) characters (eg, emojis), replaced all user name mentions with “__username__,” all email addresses with “__email__,” and all numbers with “__number__.” We also reduced characters elongation by removing the repetition of a character after the second occurrence eg, “greeeeeeat” would become “greeat”), and lowercased all sentences.

Using the preprocessed sentences and aiming at maximizing the informativeness and the variability of the texts, we limited the number of tweets any user could contribute to 5 and discarded sentences shorter than 20 characters in length, retweets, tweets not written in English, sentences containing keywords related to marketing campaigns (for this we created a list built heuristically using 5 words commonly related to marketing campaigns: “buy,” “cheap,” “online,” “pharmacy,” “price”), and also discarded sentences including URLs.

To discard possibly duplicated sentences, we stored 40-character long substrings appearing in the chosen sentences and searched for these substrings in the candidate sentences keeping only the messages not containing them. For each chosen sentence, we only stored one substring composed of a maximum of 40 characters (less for sentences shorter than 60 characters in length), extracted from the character in position 20th onwards. This decision was driven by the observation that there were a number of tweets conveying the same information using minor rewording for the sentences, making them unique. In this scenario, discarding the sentences replicating information contributes to increase the information diversity.

This strategy aims at further increasing the variability of the texts by filtering out similar messages, and in case of selecting the message “Lisinopril is used for treating high blood pressure alone or with other medicines. Other names for this medication. Acecomb, Acelisino” by extracting the characters in position 20 to position 40 (“or treating high blood pressure alone or”), we are able to discard possible duplicated sentences such as “lisinopril and hctz 20 mg 25 mg—national institutes of...lisinopril is used for treating high blood pressure alone or with other,” and similarly the system is also able to discard the sentence “Jun 29, 2015...Active Ingredient: Lisinopril. Prinivil is used for treating high blood pressure alone or with other medicines. Other.” This strategy also showed its usefulness when applied to PubMed sentences as observed in the substring “mg oral granules are bioequivalent to s” appearing twice in the same article, first in the abstract (“Sandoz montelukast 4 mg oral granules are bioequivalent to Singulair 4 mg mini oral granules, with a similar safety profile *.* ”), and also in the discussion (“The current study has clearly demonstrated that Sandoz montelukast 4 mg oral granules are bioequivalent to Singulair mini 4 mg oral granules in terms of the rate and extent of absorption of each formulation *.* ”), thereby showing that this method can help in reducing the amount of duplicated information.

Out of the resulting sentences, we automatically selected 6000 sentences each for both Twitter and PubMed, which we extracted in a round-robin fashion aiming at a balanced sample of the drug mentions.

We were interested in finding which sentences would be of interest, for which we divided the main task in two phases. During the first phase, both annotators were requested to perform a sentence level annotation to extract 1000 positive sentences (ie, the sentences mentioning drugs, symptoms, and diseases related to the drug effects in humans) out of the 6000 sentences. In the second phase, the annotators would use the annotation guidelines to identify the entities and relations appearing in the 1000 sentences identified during the first phase.

The aim of this pipeline is to filter the most informative sentences, discarding those sentences that are prone to include information that is not of high relevance for pharmacovigilance studies.

**Figure 1:**
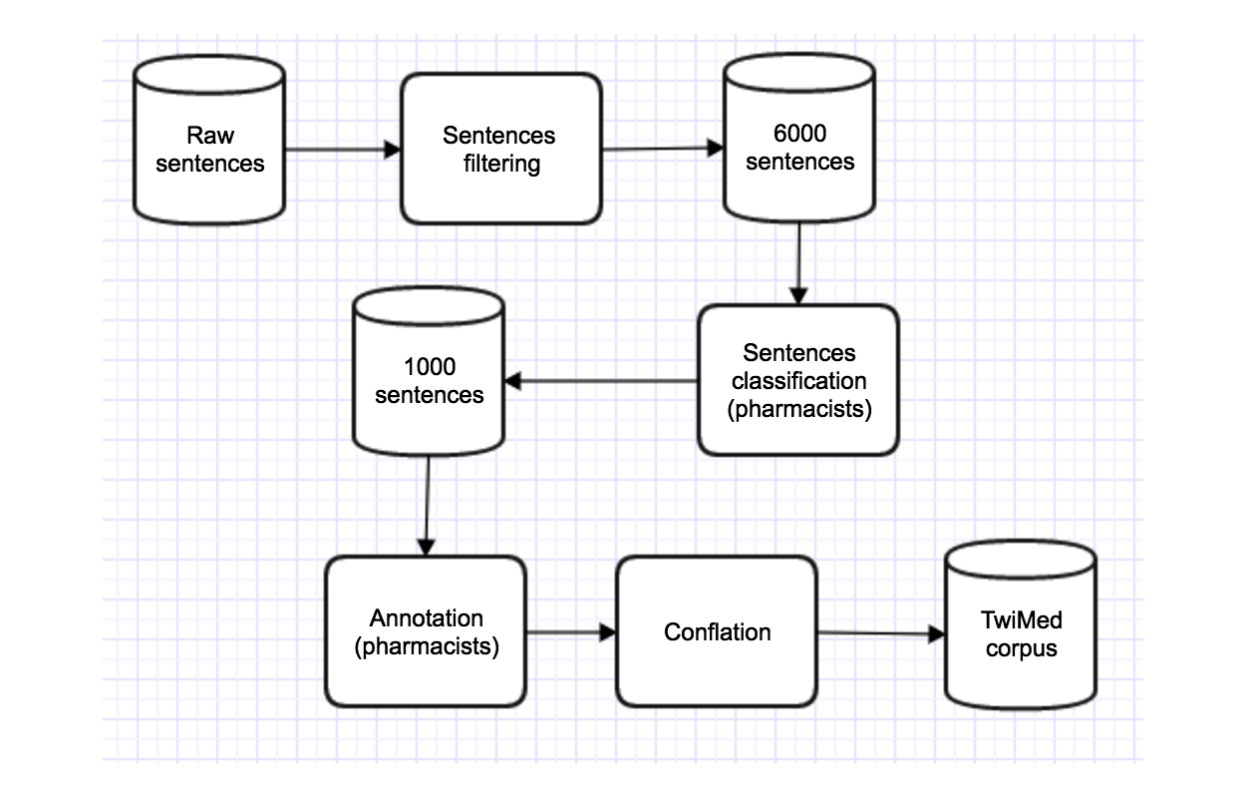
Annotation pipeline. The initial number of raw sentences differed between twitter (165,489 tweets) and PubMed (29,435 sentences).

Figure 1 shows the pipeline used to filter, classify, and annotate the sentences. Despite the difference in the initial number of raw sentences we had from Twitter and PubMed (165,489 tweets and 29,435 PubMed sentences), the steps described in the figure provided the same number of sentences at the end of each process.

### Selecting the Annotators

We identified 6 people who were willing to contribute to the task and prepared a test to identify the best candidates. We provided them with 20 sentences from Twitter and 20 sentences from PubMed obtained from the 6000 sentences we had previously filtered, and their task was to identify the sentences containing a mention to a drug and a related disease or symptom. The 6 candidate annotators had different backgrounds: one of them was a native English speaker, three of them were pharmacists, and the last two of them were active social media users. Except for the native English speaker, the rest of the annotators were native Spanish speakers able to read English texts. Although the pharmacists are not referred to as “active social media users,” the three of them were millennials who used social media networks to some extent.

### Annotation

The annotation guidelines were prepared after reviewing existing guidelines used in other pharmacovigilance projects. In the ADE corpus guidelines [10], the researchers annotated the drugs, adverse effects, dosages, and the existing relations between these elements, whereas in the meta-knowledge annotation of bio-events [11], the researchers followed a slightly different approach and focused on different “dimensions” of the biomedical events. Those dimensions can be thought as attributes of those events as these dimensions are the knowledge type, the level of certainty, the polarity, the manner, and the source of the annotated event.

Similarly, to the annotation of the ADE corpus, the Arizona disease corpus (AZDC) annotation guidelines [41] focused on the annotation of the diseases, also covering syndromes, illnesses, and disorders. Another document consulted to prepare the first draft of our annotation guidelines was the shared annotated resources (ShARe) or Conference and Labs of the Evaluation Forum (CLEF) eHealth 2013 shared task I [12], where the authors annotated disorders using the concept unique identifier (CUI), also clarifying that a disorder is understood as “any span of text that can be mapped to a concept in the SNOMED-CT terminology, which belongs to the Disorder semantic group,” clarifying that the Disorder semantic group should include “congenital abnormalities,” “diseases or syndromes,” and “signs and symptoms” among others.

Other supporting document used to prepare the annotation guidelines was the annotation guidelines for the drug-drug interaction (DDI) corpus [9], where the authors focused on the annotation of a number of entities such as drugs approved for human use, brand names for approved drugs, drugs that have not been approved for human use, and different drug groups. These guidelines also describe the annotation for different types of relations existing between the entities: “advice,” “effect,” “mechanism,” or “other.”

The details on the resulting corpora produced by the researchers using the aforementioned annotation guidelines can be found in Table 1.

**Table 1:** Details on the resulting corpora produced by the researchers who used the guidelines we reviewed.

Corpus name	DDI^a^corpus	ADE^b^corpus	AZDC^c^corpus	ShARe^d^ or CLEF^e^ eHealth 2013 Task I
Annotated entities	Pharmacological substances	Drug, adverse effects, dosages	Diseases	Disorders
Annotated relations	Drug-drug interactions	Drug-adverse effect, drug-dosage	-	-
Texts origin	DrugBank and MEDLINE	MEDLINE	PubMed abstracts	Clinical notes
Number of documents	1025	2972	794 (2775 sentences)	200
Number of annotators	2	3 (after automatic annotation)	2 (after automatic annotation)	2
Availability	Free use for academic research	Free	Free	Upon request
Annotation Tool	Brat	Knowtator	In-house tool	Knowtator

^a^DDI: drug-drug interaction.

^b^ADE: adverse drug event.

^c^AZDC: Arizona disease corpus.

^d^ShARe: shared annotated resources.

^e^CLEF: Conference and Labs of the Evaluation Forum.

As shown in Table 1, for the annotation of these corpora, researchers have used tools such as Brat [42] and Knowtator [43]. In our case, Brat tool was chosen after taking into account that it is a Web-based annotation tool that eases key elements of the annotation process.

The use of the mentioned guidelines eased the task of generating the first draft of our guidelines, and allowed us to identify which were the entities, relations, and attributes to be annotated. This first draft was then used by three external annotators with a background in computer science who annotated a small set of PubMed and Twitter sentences. During that first annotation period, we had daily meetings after each annotation session and refined the guidelines upon the discrepancies we found and the questions raised by the annotators. Those comments and question in combination with the information we found in the existing guidelines was used to provide the annotators with an updated version of the guidelines for the next annotation session.

After 2 weeks and 6 annotation sessions, the number of discrepancies was reduced to a minimum and no more questions were raised, leading us to agree on freezing the guidelines so that these would be used as they were.

The final version of the guidelines used in our study includes three different entities: (1) Drug: any of the marketed medicines that appears in the SIDER database [44], which is also listed in the closed set of drugs we provided to the annotators, (2) symptom: any sign or symptom contained in MedDRA [45] ontology, and (3) disease: any disease contained in MedDRA ontology.

The use of SIDER, which contains information on marketed medicines extracted from public documents and package inserts, and MedDRA, a medical terminology dictionary aimed at easing the annotators’ task by providing them two well-known resources to provide the annotated entities with a standardized concept identifier. We believe the fact that those resources are of common use in the research community, and combined with the current trend to map concepts in these databases to concepts in other resources [46,47], provides an important element toward TwiMed corpus reuse.

- Polarity: Used to indicate whether the entity was negated or not. The negation had to be a linguistic negation (“not,” “don’t”...).
- Person: Used to indicate whether the entity was affecting the “1st,” “2nd,” “3rd” person, or whether there was no information. This attribute was based on the original sender.
- Modality: Used to indicate whether the entity was stated in an “actual,” “hedged,” “hypothetical,” or “generic” way.
- Exemplification: Used to indicate whether the entity was presented using an example or a description. This attribute was only to be used when the entity was presented through an exemplification.
- Duration: Used to indicate whether the entity’s lasting span was “intermittent,” “regular,” “irregular,” or not stated. In the case of drugs, this attribute referred to the time span when the drug had been taken.
- Severity: Used to indicate whether the seriousness of an entity was “mild,” “severe,” or not stated. This was the only attribute that did not apply to drugs.
- Status: Used to indicate whether the duration of the entity was “complete,” “continuing,” or not stated. In the case of drugs, this attribute referred to the time span when the drug was perceived as having effect.
- Sentiment: Used to indicate whether the entity was perceived as “positive,” “negative,” or “neutral.”
- Entity identifier: Used to indicate the CUI for that entity. This was the only attribute that had to be filled for all annotated entities. For this attribute we provided a list of allowed values, and used the value “−1” (not found) for entities whose CUI would not be present in the list.

The list of attributes was decided based on the combination of elements noticed to be annotated in pharmacovigilance studies using formal texts (eg, “duration” or “modality”), as well as in pharmacovigilance studies using informal texts (eg, “polarity” or “sentiment”).

- Reason-to-use: Used to represent the relation appearing when a symptom or disease leads to the use of some drug.
- Outcome-positive: Used to represent the relation between a drug, and an expected or unexpected symptom or disease appearing after the drug consumption. The outcome had to be positive.
- Outcome-negative: Used to represent the relation between a drug, and an expected or unexpected symptom or disease appearing after the drug consumption. The outcome had to be negative.

These elements are further explained in the annotations guidelines that are shared in the Multimedia Appendix 2.

Once the guidelines were ready and the annotators were chosen, we preprocessed the sentences before presenting them to the annotators by replacing the existing emojis with a string describing each character, and discarded other non-ASCII characters. We also decided not to lower case the sentences as we thought that would ease the annotator’s task to detect some sentiments and disambiguate acronyms. Besides these changes, the preprocessing strategy is the same we described in the “data selection” section.

To compare the annotations produced by the experts, we focused on both the “type” assigned to the entity (ie, disease, drug, or symptom) and also on the offsets for that entity. Taking that into account, we decided to compute the results when using relaxed constraints and strict constraints. In the case of using relaxed constraints, we say that the entity annotated by both annotators is a match if the type for the entity matches between annotations and the spans of those annotations have some overlap. In the case of using strict constraints, the match would happen if the type in both annotations matches and the spans for the annotated entities have the same offsets. Discontinuous annotations were allowed and taken into account when computing the matches, which means that in case of using strict constraints, all the spans taking part on the entity’s annotation should be the same.

We measured the level of agreement between the annotations produced by our experts following the inter annotator agreement (IAA) measure in the CLEF corpus [48]. This IAA metric is reported to approximate the kappa score [48], and to be more suited for this case [49]:

IAA=matches/(matches+nonmatches)

In our case matches accounts for the total number of token matches for which both annotators agreed, and matches + nonmatches counts all annotations performed by the annotator being evaluated.

## Results

### Data Selection

Out of the 6000 selected sentences each for both Twitter and PubMed that we extracted, we observed differing sample frequencies of each drug. In both Twitter and PubMed, some drugs attracted more attention than others, although in the case of Twitter, temporal variability is a known fact [26] that has to be taken into consideration.

We found that the frequency of the drugs in the extracted sample had no correlation between Twitter and PubMed (Spearman rho=.03), as shown in Table 2.

**Table 2:** Total number of sentences for each drug name in Twitter and PubMed.

Drug name	# Tweets	# Sentences in PubMed
Bevacizumab	69	239
Buprenorphine	363	244
Carbamazepine	74	239
Ciprofloxacin	81	250
Citalopram	331	251
Cortisone	344	231
Destroamphetamine sulphate	373	19
Docetaxel	34	246
Duloxetine	242	241
Fluoxetine	344	238
Fluvoxamine maleate	13	204
Lamotrigine	168	242
Lisdexamfetamine	348	84
Lisinopril	56	147
Melphalan	2	234
Methylphenidate hydrochloride	349	112
Modafinil	287	10
Montelukast	71	239
Olanzapine	190	248
Paroxetine	365	249
Prednisone	350	249
Quetiapine	339	247
Rupatadine	1	45
Sertraline	343	236
Tamoxifen	122	238
Topiramate	133	231
Trazodone	206	70
Triamcinolone acetonide	14	253
Venlafaxine	326	238
Ziprasidone	62	226

### Selecting the Annotators

To evaluate the annotator’s performance, we used a gold standard set of labels that we generated obtaining the majority vote from the results we received from the 6 annotators and the annotations produce by the first author of the paper, also giving more weight to the pharmacists’ annotations in PubMed and to social media users’ annotations in Twitter. That is, when there were clear differences between the annotations provided by the contributors with the higher weights and the rest of the annotators, we took the former annotations into account.

As can be seen in Table 3, one pharmacist scored the best result, 87.5% agreement with the gold standard data (35 out of 40 sentences were correctly labelled).

**Table 3:** Agreement with gold standard data during the annotator selection phase. We compared the results from 2 very active social media users, one native English speaker and 3 pharmacists. We indicate between brackets the time it took to complete the annotation for that dataset (time in min).

Annotator	Twitter (min)	PubMed (min)	Total (min)
Social1	0.70 (9)	0.80 (10)	0.75 (19)
Social2	1.00 (8)	0.70 (7)	0.85 (15)
Native speaker	0.85 (6)	0.50 (6)	0.67 (12)
Pharmacist1	0.90 (8)	0.85 (7)	0.87 (15)
Pharmacist2	0.70 (11)	0.80 (9)	0.75 (20)
Pharmacist3	0.50 (15)	0.70 (15)	0.60 (30)

Those results were in line with our expectations as social media users got the best scores in social media texts, and the best scores in PubMed texts were obtained by the pharmacists. However, we were very surprised by the low scores obtained by Pharmacist3 and the native English speaker. We followed up with them discovering that Pharmacists3 had some trouble understanding the samples because of those being written in English language (it was also evidenced in the time it took her to complete the task). In the case of the native English speaker, he reported that he was not an active social media user and requested further information on the set of tweets as he found those texts to be hard to understand. Overall, we discovered the native English speaker was too cautious when indicating which sentences were positive cases as he annotated 7 sentences as positive out of the 40 sentences (the gold standard data had 16 sentences tagged as positive sentences), whereas the rest of the annotators indicated 13-18 sentences were positive (Pharmacist3, who obtained the lowest score, was above that range as she annotated 24 sentences as positive).

We decided to hire Pharmacists1 as she scored the best results, and out of Social1, Social2, and Pharmacist2, we decided to hire Pharmacist2 taking into account that the resulting corpus would require annotation at entity level for which Pharmacist2’s in-domain knowledge would be very valuable.

### Annotation

Once the 2 pharmacists competed the annotation at sentence level, we focused on the entity level annotation targeting at the diseases, drugs, and symptoms. The results for Twitter and PubMed are shown in Tables 4 and 5, using the relaxed constraints and strict constraints strategy described in the Methods section.

**Table 4:** Detail of annotations in Twitter. The first column shows the element being evaluated. Columns 2-5 show the inter annotator agreement scores of pharmacist 1 (Ph1) and pharmacist 2 (Ph2) using relaxed and strict constraints. Columns 6 and 7 show the number of elements annotated by each pharmacist. Columns 8 and 9 show the number of matching elements between pharmacist’s annotations using relaxed and strict constraints.

Annotated element	Ph1 (relaxed constraints)	Ph2 (relaxed constraints)	Ph1 (strict constraints)	Ph2 (strict constraints)	#Ph1	#Ph2	#Matches (relaxed constraints)	#Matches (strict constraints)
Drug	97.39	98.72	93.52	94.80	1111	1096	1082	1039
Disease	50.86	91.47	46.12	82.95	464	258	236	214
Symptom	77.23	76.71	54.21	53.84	1164	1172	899	631
Outcome-negative	63.27	75.19	43.02	51.12	795	669	503	342
Outcome-positive	11.01	40.00	8.26	30.00	109	30	12	9
Reason-to-use	55.82	60.18	44.66	48.14	842	781	470	376
Duration	46.37	8.96	39.11	7.56	248	1283	115	97
Exemplification	10.11	64.77	3.37	21.59	564	88	57	19
Modality	56.92	30.58	49.57	26.63	585	1089	333	290
Person	72.56	58.55	60.21	48.58	1709	2118	1240	1029
Polarity	76.06	52.43	53.52	36.89	71	103	54	38
Sentiment	72.48	19.46	60.92	16.36	476	1773	345	290
Severity	64.18	19.59	44.03	13.44	134	439	86	59
Status	59.41	22.07	45.94	17.07	542	1459	322	249

**Table 5:** Detail of annotations in PubMed. The first column shows the element being evaluated. Columns 2-5 show the inter annotator agreement scores of pharmacist 1 (Ph1) and pharmacist 2 (Ph2) using relaxed and strict constraints. Columns 6 and 7 show the number of elements annotated by each pharmacist. Columns 8 and 9 show the number of matching elements between pharmacist’s annotations using relaxed and strict constraints.

Annotated element	Ph1 (relaxed constraints)	Ph2 (relaxed constraints)	Ph1 (strict constraints)	Ph2 (strict constraints)	#Ph1	#Ph2	#Matches (relaxed constraints)	#Matches (strict constraints)
Drug	95.20	97.90	86.23	88.67	1271	1236	1210	1096
Disease	64.18	95.22	53.41	79.23	1086	732	697	580
Symptom	85.13	60.59	70.61	50.26	558	784	475	394
Outcome-negative	60.97	64.86	50.35	53.56	433	407	264	218
Outcome-positive	56.25	32.73	43.75	25.45	32	55	18	14
Reason-to-use	62.87	77.39	47.10	57.98	1535	1247	965	723
Duration	52.17	9.38	48.70	8.75	115	640	60	56
Exemplification	0.64	50.00	0.32	25.00	311	4	2	1
Modality	74.23	50.52	64.60	43.96	1370	2013	1017	885
Person	63.93	77.18	56.08	67.70	1439	1192	920	807
Polarity	25.00	22.22	25.00	22.22	16	18	4	4
Sentiment	33.33	1.96	22.22	1.31	9	153	3	2
Severity	42.22	33.33	37.78	29.82	45	57	19	17
Status	53.85	2.52	53.85	2.52	26	555	14	14

By focusing on the results appearing in Tables 4 and 5, we see that the agreement for the drugs in Twitter and PubMed is very high, which was expected, given our sampling strategy, although for diseases and symptoms the agreement score decreases noticeably in both Twitter and PubMed.

When comparing the results for the relations (outcome-negative *,* outcome-positive, and reason-to-use), we saw low levels of agreement, having Twitter lower results in all cases. Analyzing the number of annotations it was clear that the use of outcome-positive relation varied considerably between annotators, contributing to the low scores.

The attributes “person” (in PubMed), “modality” (in both PubMed and Twitter), “polarity,” and “sentiment” (in Twitter) were the ones obtaining the best scores. On the other hand, the attribute “exemplification” (in both PubMed and Twitter), “sentiment,” “polarity” (in PubMed), and “duration” (in Twitter) were very prone to disagreements as these scores were the lowest in Tables 4 and 5.

**Figure 2:**
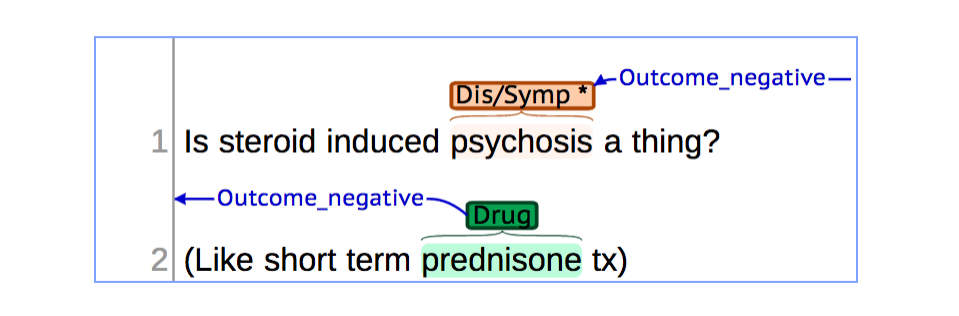
Sample with the annotation of a drug, a disease and the relation between these concepts in a sentence from Twitter.

By analyzing the discrepancies in the annotations, we discovered that the distinction between disease and symptom entities, although theoretically clear, was hard to disambiguate in a number of sentences. We can see that in the tweet “Is steroid induced psychosis a thing? (Like short term prednisone tx)” (see Figure 2), psychosis could be identified as a symptom [50,51] or as a disease [52]. Similarly, the entity comorbid obesity found in the PubMed sentence “The present case report of topiramate’s effect on comorbid obesity,” could be also understood as both a symptom and a disease [53]. Interestingly, we observed in those examples that even if the chosen type of entity (disease and symptom) was different, the annotators agreed on the chosen CUI.

In the case of relations, we discovered that both outcome-positive relation and reason-to-use relation were confounded in some cases. One example from Twitter is the sentence “How about trazodone, so I can just feel a little funny and then knock out and have the best sleep of my life,” where the drug, trazodone, and the symptom, the best sleep of my life, were annotated as such by both annotators, although one annotator indicated the relation between these entities was an outcome-positive relation whereas the other annotator marked it as a reason-to-use relation. The same observation was seen in PubMed sentences as in “Because fatigue is a frequent symptom of depression and there is some evidence that treatment with an antidepressant improves fatigue in patients with fibromyalgia, we hypothesized that the antidepressant fluvoxamine might improve fatigue related to PBC and PSC.” In this sentence, the drug (fluvoxamine) and the symptom (fatigue) were correctly identified, same as the existing relation between the entities, but the chosen type of relation was different. This observation, combined with the fact that outcome-positive relation was the least used type of relation, helps in understanding the causes for the low inter annotator agreement score.

Given the similarities between those concepts and the disagreements that we detected, we evaluated the inter annotator agreement score when conflating the concepts disease and symptom under “disease or symptom” concept. We also grouped together outcome-positive and reason-to-use relations under “benefit” relation. The use of those categories produced a noticeable improvement in the IAA scores. This strategy also improved the agreement scores for most of the attributes as can be seen in Table 6 (for Twitter), and Table 7 (for PubMed).

**Table 6:** Detail of annotations in Twitter using the conflation strategy. The first column shows the element being evaluated. Columns 2-5 show the inter annotator agreement scores of pharmacist 1 (Ph1) and pharmacist 2 (Ph2) using relaxed and strict constraints. Columns 6 and 7 show the number of elements annotated by each pharmacist. Columns 8 and 9 show the number of matching elements between pharmacist's annotations using relaxed and strict constraints.

Annotated element	Ph1 (relaxed constraints)	Ph2 (relaxed constraints)	Ph1 (strict constraints)	Ph2 (strict constraints)	#Ph1	#Ph2	#Matches (relaxed constraints)	#Matches (strict constraints)
Drug	97.39	98.72	93.52	94.80	1111	1096	1082	1039
Disease or symptom	82.25	93.64	61.36	69.86	1628	1430	1339	999
Outcome-negative	67.30	79.97	46.29	55.01	795	669	535	368
Benefit	68.14	79.90	52.37	61.41	951	811	648	498
Duration	50.00	9.66	41.94	8.11	248	1283	124	104
Exemplification	10.11	64.77	3.37	21.59	564	88	57	19
Modality	64.44	34.62	54.53	29.29	585	1089	377	319
Person	77.30	62.37	63.96	51.61	1709	2118	1321	1093
Polarity	80.28	55.34	57.75	39.81	71	103	57	41
Sentiment	75.00	20.14	62.61	16.81	476	1773	357	298
Severity	67.16	20.50	47.01	14.35	134	439	90	63
Status	61.81	22.96	48.15	17.89	542	1459	335	261

**Table 7:** Detail of annotations in PubMed using the conflation strategy. The first column shows the element being evaluated. Columns 2-5 show the inter annotator agreement scores of pharmacist 1 (Ph1) and pharmacist 2 (Ph2) using relaxed and strict constraints. Columns 6 and 7 show the number of elements annotated by each pharmacist. Columns 8 and 9 show the number of matching elements between pharmacist’s annotations using relaxed and strict constraints.

Annotated element	Ph1 (relaxed constraints)	Ph2 (relaxed constraints)	Ph1 (strict constraints)	Ph2 (strict constraints)	#Ph1	#Ph2	#Matches (relaxed constraints)	#Matches (strict constraints)
Drug	95.20	97.90	86.23	88.67	1271	1236	1210	1096
Disease or symptom	91.91	99.67	74.21	80.47	1644	1516	1511	1220
Outcome-negative	81.52	86.73	65.82	70.02	433	407	353	285
Benefit	77.41	93.16	56.86	68.43	1567	1302	1213	891
Duration	53.91	9.69	50.43	9.06	115	640	62	58
Exemplification	0.64	50.00	0.32	25.00	311	4	2	1
Modality	83.43	56.78	71.39	48.58	1370	2013	1143	978
Person	71.58	86.41	62.13	75.00	1439	1192	1030	894
Polarity	43.75	38.89	43.75	38.89	16	18	7	7
Sentiment	33.33	1.96	22.22	1.31	9	153	3	2
Severity	53.33	42.11	46.67	36.84	45	57	24	21
Status	53.85	2.52	53.85	2.52	26	555	14	14

## Discussion

### Principal Findings

We produced a corpus of documents obtained using a very similar pipeline for both Twitter and PubMed. All the documents were filtered and double annotated by the same experts (pharmacists).

TwiMed corpus shows that the drugs appearing less often in Twitter are those used to treat cancer (“docetaxel,” “bevacizumab,” “tamoxifen”) and epilepsy (“topiramate,” “carbamazepine”). On the other hand, the drugs used to treat attention deficit hyperactivity disorder (“modafinil,” “destroamphetamine sulphate,” “lisdexamfetamine”) are the drugs having the most mentions in Twitter, whereas at the same time those are the drugs having the least mentions in PubMed sentences. This fact evidences that public concerns are not always aligned with the interests of the scientific community, and potential areas of research may emerge from those findings to understand the reasons and related outcomes.

In our analysis, we observed a much higher balance for the drug mentions in PubMed than in Twitter. Factors such as the demographics of the user base in Twitter [54], or the time when the messages were gathered [26] are elements that should be studied in detail to measure their correlation with the different distribution of drug mentions.

Given our main goal was to create a corpus covering mentions of drugs used to treat a number of different conditions, we applied a set of controlling mechanisms to extract the data from Twitter and from PubMed, and researchers should be aware that the drugs in this corpus have different distribution in the original sources of information than the distributions presented here, and following quantitative studies may be needed to understand those differences.

We believe the reason for the high agreement in the annotation of drugs is the use of a closed set of drug names that both annotators knew beforehand. We can see, however, that there is a lower level of agreement for the annotation of symptoms and diseases and the main reason would be that the annotators had to identify these mentions, and there is an open list of entities that can be found in the texts, not to mention that these entities could be presented using an exemplification. In addition, in some contexts a disease can be considered as a symptom, and the short nature of the sentences can act as a factor in confounding the nature of these entities. Similarly, more subjective concepts such as the duration attribute or the exemplification attribute show a low level of agreement probably because the annotators had to interpret these elements by themselves and in some cases a certain level of subjectivity led the decision.

Researchers should be aware that this corpus is not devised to capture everything about the selected set of 30 drugs, and there are a number of drug names appearing in the selected set of sentences which were not annotated because these were not included in the target set of drugs. Similarly, there are DDIs and other relations that the corpus does not include because of our constraints. However, we believe the provided sample can help in training NLP systems to capture more information. Nonetheless, we provide a set of semantically correct annotations that can be used in NLP studies.

Our annotation also confirmed that there is lower agreement in the annotation of tweets than in the annotation of PubMed sentences, showing the noisy nature of Twitter [13]. Moreover, when applying our conflation strategy aimed at resolving disagreements, we observed that these differences still remained.

We noticed that a number of those disagreements were caused when confounding “diseases” and “symptoms.” Similarly, acronyms appearing in documents from PubMed tend to be explained the first time they are presented, which does not necessarily have to be when the drugs and related symptoms and diseases are discussed. In our case we allowed the annotators to access the full articles during the annotation process to reduce the impact of this problem. Nonetheless, we believe the use of acronyms is a potential source of confusion in texts where the context is scarce, and this potential problem should be handled.

**Figure 3:**
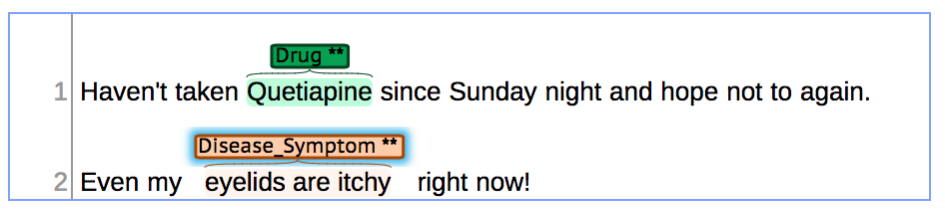
Sample of an annotation where “duration” and “exemplification” attributes are used.

Additionally, other noticeable finding when using the same guidelines is the fact that disagreements appear in similar categories for both Twitter and PubMed. Figure 3 shows an example where the string “eyelids are itchy” was annotated with the duration of “regular” by one annotator (to indicate that there is a continued lasting span), whereas the other annotator chose “irregular” for the duration attribute (to indicate that there is no pattern in the lasting span).

Figure 3 also shows an example where the annotation for the attribute “exemplification” differs between annotators as the string “eyelids are itchy” was annotated as an exemplification by only one of the two annotators.

Besides PubMed-Twitter comparative studies, our corpus is of potential interest for researchers aiming at finding sentences containing information on the drugs, symptoms, and diseases. We believe this corpus can become a useful resource to discern informational sentences in the area of pharmacovigilance as other researchers can use the sentences we included in this dataset to create classifiers targeting at the correct identification of sentences reporting drug-use.

This dataset shows that for similar events coming from very different data sources, the way in which people communicate the same messages has noticeable differences. This corpus can provide useful insights to science communicators and public institutions for adapting their messages when addressing the general public so that the information can attract more attention. One of such examples would be the use of social media by official health institutions, where most of the messages are more formal than average social media messages, as a mean to reach a wider audience during health promotion and disease prevention campaigns as the wording may affect the impact of the messages.

We believe combining the information contained in scientific reports, of high quality and very trustworthy, together with the information coming from social media messages, which is global, has a high volume, and is up-to-date, should be taken into account when building pharmacovigilance systems. We hope this corpus can help researchers interested in combining the potential of those data sources.

### Conclusions

We have presented a pharmacovigilance corpus that, to our knowledge, is the first corpus that allows researchers to perform direct comparative studies toward understanding the differences between drug reports in Twitter and PubMed.

Our corpus contains annotations for drugs, symptoms, and diseases; their attributes (polarity, person *,* modality, exemplification, duration, severity, status, sentiment); and the relations between the annotated entities (reason-to-use, outcome-negative, and outcome-positive).

We also identified the source of a number of disagreements for the annotated entities and relations, and proposed a conflation strategy to resolve those discrepancies. That approach resulted in higher agreement scores for most entities and relations.

We hope that given the comprehensive set of drug names and the annotated entities and relations included in this corpus, it can become a standard resource to compare results from different pharmacovigilance studies, especially in the area of NLP as it can help in training to recognize the entities and relations in the texts. Similarly, this corpus can help in comparing the performance of NLP tools across the 2 different linguistic registers (formal and informal).

In summary, we present a comparable corpus for pharmacovigilance studies and the annotation scheme we devised. This work is presented to the research community in the belief that such resources can help in this rapidly growing area.

The corpus we release, available as Multimedia Appendix 1, contains the annotations for all the entities, relations, and attributes where both annotators agreed. Additionally, we provide the tools to obtain the raw tweets used in the annotation, to comply with Twitter’s terms of service [55], and we also provide the tools to preprocess the raw sentences from both Twitter and PubMed to reuse the released annotations.

## Appendix

Multimedia Appendix 1 TwiMed corpus.

Multimedia Appendix 2 Annotation guidelines used to prepare TwiMed corpus.

## Abbreviations

ADE: adverse drug events
ASCII: American standard code for information interchange
CLEF: Conference and Labs of the Evaluation Forum
CUI: concept unique identifier
DDI: drug-drug interaction
IAA: inter annotator agreement
NLP: natural language processing
ShARe: shared annotated resources
