# Supplementary material for: TwiMed: Twitter and PubMed Comparable Corpus of Drugs, Diseases, Symptoms, and Their Relations
Source: JMIR Public Health Surveill. 2017 May 3;3(2):e24. doi: 10.2196/publichealth.6396 (PMC5438461; doi:10.2196/publichealth.6396)
Supplement: Multimedia Appendix 1 [file jmir_v3i2e24_app1.zip › TwiMed-master/Guidelines_TwiMed.pdf]

Guidelines for Drug-Disease-Symptom annotation in Twitter and PubMed texts.

VERSION 0.9 (4th-Sept-2015)

Introduction

The goal of this project is to create an annotated corpus of symptoms, diseases, and drugs mentions in sentences taken from PubMed articles and from tweets.
In the rest of the document we will refer to the symptoms, diseases, and drugs using their capitalized form (SYMPTOMS, DISEASES, and DRUGS) when we talk about the generic entities that are to be annotated.
For this study we focus on a closed set of DRUGS (Table A1, in the appendix), although the study is not limited to them, and any mention of any DRUG in our closed list of DRUGS should be annotated.
This document provides a definition of SYMPTOM, DRUG, and DISEASE, the relations between these entities, and the guidelines to be followed during the annotation.
In case there is some question not covered in this document, please send an e-mail to: nestoralvaro@nii.ac.jp .
In these guidelines we describe each one of the 3 types of entities (DRUGS, SYMPTOMS, and DISEASES), the attributes of the entities, and the 3 types of relations (reason to use, outcome-positive, outcome-negative) that are to be annotated.

Annotation of entities

An entity can be a single word such as "*tiredness*" as it appears in the sentence "*the patient was experiencing tiredness*", or a span of text such as "*could not move from the couch*" obtained from the sentence "*I worked out so hard that when I got back home I could not move from the couch*".
Both entities refer to the concept "tiredness symptom" (MEDDRA Code 10043890).
We provide a list of entities, the definition of each one and an example in Table 1.
The only DRUGS to be annotated are those appearing in Table A1.
In the following examples **DRUGS** are highlighted in green, **SYMPTOMS** in blue, and **DISEASES** in red.

Entity	Definition	Example
**DRUG**	Any of the marketed medicines that appears in the SIDER database (http://sideeffects.embl.de/), which is also listed in our closed set of drugs (See Table A1).	The prescription included **Lexapro**.
**SYMPTOMS**	Any sign or SYMPTOM contained within the MedDRA ontology (http://bioportal.bioontology.org/ontologies/MEDDRA).	**Adderall** kept me **focused**.
**DISEASE**	Any DISEASE contained within the MedDRA ontology. (http://bioportal.bioontology.org/ontologies/MEDDRA).	The patient suffered from **sleep deprivation** without **trazodone**.

Table 1: Entities to be annotated.

Annotation of attributes

The entities (DRUGS, SYMPTOMS and DISEASES) have some attributes that will be annotated to

clarify some concepts.
We provide a list of attributes for the entities, the definition of each one, the values each attribute can take, and an example in Table 2. Some attributes have **default values (in bold and highlighted in the table)** which will be used when no attribute is chosen.
In the following examples **DRUGS** are highlighted in green, **SYMPTOMS** in blue, and **DISEASES** in red.

Attribute	Definition	Values	Examples
**Polarity**	Indicates whether the entity is negated or not. The negation has to be a linguistic negation (“not”, “don’t”...).	• **Positive**: The entity is not negated. Default value.<br>• **Negative:** The mention of the entity is negated.	*“I took **prozac** and now I don’t have a **headache**”*<br>**Prozac**: polarity=positive (left blank)<br>**Headache**: polarity=negative
**Person**	Indicates whether the entity is affecting the “1st”, “2nd”, “3rd” person, or whether there is no information. This attribute is based on the original sender.	• **1st:** The entity is described from a “first person” point of view. The entity is directly impacting the author of the text. Relates a first hand experience.<br>• **2nd:** The entity is described from a “second person” point of view. The entity is impacting another person whom the author knows.<br>• **3rd:** The entity is described from a “third person” point of view. The entity is impacting someone not directly related with the author of the text.<br>• **Not available:** There is no clear reference to whom the entity is impacting. Default value.	*“I took **prozac** and now I don’t have a **headache**”*<br>**Prozac**: Person=1st<br>The entity is described in first person.<br>**Headache**: Person=1st<br><br>*“Hate **prozac**”*<br>**Prozac**: Person=not available (value left blank).
**Modality**	Indicates whether the entity is stated in an “actual”, “hedged”, “hypothetical” or “generic” way.	• **Actual:** These mentions have already happened or are being scheduled (without hedging) to happen. Default value.<br>• **Hedged:** These mentions include lexical ("seems", "likely", "suspicious", "possible", "consistent with"), or phrasal ("I suspect that...", "It would seem likely that") hedging. These entities are strongly implied, but, for safety, liability, or due to lack of comprehensive evidence, are not stated as a fact.<br>• **Hypothetical:** Will often follow "if" statements ("If X happens, then we’ll use Y to treat Z") or other sorts of conditionals ("Depending on the patient’s response, we might treat A	*“The patient did not report **nausea**”.*<br>**Nausea**: Modality=Actual<br><br>*“The patient may have undergone a mild stroke”*<br>**Stroke**: Modality=hedged<br><br>*“We suspect either **achalasia** or **pseudoachalasia** here”*<br>**Achalasia**: Modality= Hypothetical<br>**Pseudoachalasia**: Modality= Hypothetical<br><br>*“**Adderall** should not be taken*

		with B or with C").<br>• **Generic:** When the mention is done in a general sense. These usually occur when putting justifications of decisions, or rationales for changing care.	*with other medications.”*<br>**Adderall**: Modality= Generic
**Exemplification**	Indicates whether the entity is presented using an example or a description. Only to be used when the entity is presented through an exemplification.	• **Positive**: When an exemplification is used to present the entity.<br>• **Negative:** The entity is not presented through an example. Default value.	“***I will not be able to get up*** *unless I take my* ***Adderall***”<br>**I will not be able to get up**: Exemplification=True Indicates “lack of energy” (SNOMED ID: 248274002)<br>**Adderall:** Exemplification=Negative (value left blank).
**Duration**	Indicates whether the entity’s lasting span is “Intermittent”, “Regular”, “Irregular”. If the duration is not indicated the attribute is left empty. In the case of DRUGS this attribute refers to the time span when the DRUG has been taken.	• **Regular**: The entity has a continued lasting span.<br>• **Intermittent**: The lasting span of the entity has been recurring.<br>• **Irregular**: There is indicated that there is no pattern in the lasting span of the entity.<br>• **Not available:** When the duration is not indicated. Default value.	“*I had a strong* ***headache*** *last night, so I took* ***prozac***.”<br>***Prozac***: Duration=not available (the value will be left empty)<br>**Headache**: Duration=”Irregular”<br><br>“*I have been on* ***Prozac*** *for 5 years now*”<br>**Prozac**: Duration=”Regular”
**Severity**	Indicates whether the seriousness of an entity is “Mild”, or “Severe”. If the severity is not indicated the attribute is left empty. ***This attribute does not apply to DRUGS.***	• **Mild**: There is gentle (not acute, nor serious) severity of the entity.<br>• **Severe**: There is a grave or critical seriousness of the entity.<br>• **Not available:** When the severity of the entity is not indicated. Default value.	“*I had a strong* ***headache*** *last night, so I took* ***prozac***.”<br>**Prozac**: Severity=not available (the value will be left empty)<br>***Headache***: Severity=”Severe”
**Status**	Indicates whether the duration of the entity is “Complete”, or “Continuing”. If the duration is not indicated the attribute is left empty. In the case of	• **Complete**: If the entity is already not showing evidence of its effects.<br>• **Continuing**: If the entity is still showing evidence of its effects.<br>• **Not available:** When the status is not indicated. Default value.	“*I had a strong* ***headache*** *last night, so I took* ***prozac***.”<br>**Prozac**: Status=not available (the value will be left empty)<br>**Headache**: Status=”Completed”<br><br>“*I took* ***prozac*** *2 hours ago, but*

	DRUGS this attribute refers to the time span when the DRUG is perceived as having effect.		*it already wore off.”*<br>**Prozac**: Status =”Complete”
**Sentiment**	Indicates whether the entity is perceived as “positive”, “negative” or “neutral”. If the entity is perceived as “neutral” this attribute is left empty.	● **Positive**: The entity is referenced as something good.<br>● **Negative**: The entity is referenced as something bad.<br>● **Neutral**: There is no clear point of view towards the referenced entity. Default value	*“I had a strong **headache** last night, so I took **prozac**.”*<br>**Prozac**: Sentiment=neutral (the value will be left empty)<br>**Headache*:***<br>Sentiment=”Negative”
**Entity identifier**	Indicates the identifier for that entity.	● **XXXXXX**: The concept identifier. The database contains a set of concepts obtained as follows:<br>○ For SYMPTOMS and DISEASES the concept identifiers represent the **UMLS concept ID** for the MedDRA term.<br>○ For DRUGS the concept identifiers represent the **PubChem concept ID** referenced in SIDER database for that concept.<br>● **-1**: If there is no concept identifier for an entity this value will be “-1”. This value can not be used for drugs (if the drug is not in the list it should **NOT** be annotated)	*“I had a strong **headache** last night, so I took **prozac**.”*<br>**Prozac**: ID=“3386”<br>**Headache**: ID=”10019211”

Table 2: Attributes of the entities.

Annotation of relations

A relation represents the existing connection between two entities. In our annotations we allow 4 types of relations. DISEASES and SYMPTOMS are not related.
We provide a list of relations, the definition of each one and an example in Table 3.
In the following examples **DRUGS** are highlighted in green, **SYMPTOMS** in blue, and **DISEASES** in red.
Relations do not have any attribute.

**Relation**	**Definition**	**Example**
**Reason to use**	Represents the relation appearing when a SYMPTOM or DISEASE leads to the use of some DRUG.	**Prozac** is indicated for patients with major **depressive disorder**.

**Outcome-positive**	Represents the relation between a DRUG, and an expected or unexpected SYMPTOM or DISEASE appearing after the DRUG consumption. The outcome has to be positive.	I wish I was prescribed **adderall**, I'd **lose weight.**
**Outcome-negative**	Represents the relation between a DRUG, and an expected or unexpected SYMPTOM or DISEASE appearing after the DRUG consumption. The outcome has to be negative.	The most common adverse events reported for **fluoxetine** were **impulsivity** and **poor concentration**.

Table 3: Relations to be annotated.

It is important to notice that the annotation tool validates the origin-entity and the end-entity of each relation. This means that:

- "Reason to use" relation: Has to start on a "SYMPTOM" or a "DISEASE" and be directed towards a "DRUG".
- "Outcome-positive" relation: Has to start on a "DRUG", and be directed towards a "SYMPTOM" or "DISEASE".
- "Outcome-negative" relation: Has to start on a "DRUG", and be directed towards a "SYMPTOM" or "DISEASE".

Practical issues

In the following examples **DRUGS** are highlighted in green, **SYMPTOMS** in blue, and **DISEASES** in red.

What to annotate?

Entities

- Each mention of an entity should be annotated exactly once. Each annotation should refer to exactly one mention of the entity. All the entities should be annotated each time they are mentioned.
- Annotate mentions with morphological variations such as adjectives.
  - For instance, “hypertensive” is annotated as “hypertension.”.
  - Hashtags, whenever present, will be included in the annotation span.
    - In the sentence “*I had a terrible* ***#headache***” the concept to be annotated is **#headache** (including the hashtag)
- Synonyms or descriptions for SYMPTOMS and DISEASES should be annotated.
  - Example: “*I Took* ***Adderall*** *and now I'm gonna be* ***up for hours***”
    - “**up for hours**” should be annotated as a synonym of “**Sleeplessness**” (notation “10041017” in MEDDRA)
- The annotations should only include the entity mention, keeping it as specific as possible, and annotate the most specific entity mentions and select the best-matching Concept ID from SIDER database (for DRUGS) or MedDRA ontology (for SYMPTOMS and DISEASES) .
  - For instance, the complete phrase “**partial seizures**” (ID: 10061334) should be preferred over “**seizures**” (ID: 10039910) as it is more specific.
  - If present, the mention span should include terms such as disease, syndrome, disorder, infection.
- Mentions of cancer, tumour, neoplasm, or infection, and other generic mentions to DISEASES/SYMPTOMS additional information, can be annotated, although it may happen that the identifier for that concept is not contained in the list of concepts.
  - In this case the ID for the concept would be “**-1**”
- An entity could be an acronym.
  - A long form, short form pair should be annotated as two mentions. Example: "***Attention deficit hyperactivity disorder*** *(**ADHD**)*". In this case "**Attention deficit hyperactivity disorder**” and “**ADHD**" should be annotated separately.
- This study is focused in a closed set of DRUGS (Table A1).
  - That list of DRUGS also includes the brand names for these DRUGS.
    - Any mention of any of this DRUGS (including the brand names) has to be **always** annotated.
  - Those drugs have different brand names and trade names. These variants have to be annotated too.
    - For example, the table contains “**Adderall**”, but “**Adderall XR**” and it should be annotated (using the DRUG identifier for **”Adderall”**, 3007)
- Lists and co-ordinations are phrases which mention multiple entities in a complex way. A simple illustrative example is “***breast*** *and* ***ovarian cancer***”, which refers to the entities “**breast cancer**” and “**ovarian cancer**”.
  - These constructs often overlap or do not explicitly mention some terms.
  - As the tool allows discontinuous annotations each entity should be annotated one time. One annotation would be “**breast cancer**” and the second annotation would be “**ovarian cancer**”.
- A retweet is a re-posting of someone else's Tweet. In this case the tweet will be considered

as if the user re-posting it would be author of the tweet. Retweets are indicated by the string "RT" at the beginning of the message.

- Example: "*RT I took **prozac** and now I don't have a **headache***"
  - This example is a retweet of "*I took **prozac** and now I don't have a **headache***", so it would be annotated as if it were "*I took **prozac** and now I don't have a **headache***"
    - **Prozac**: Person=1st
      - The entity is described in first person.
    - **Headache**: Person=1st
      - The entity is described in first person.

- There are some cases when DRUGS/SYMPTOMS/DISEASES are used as an indicator of other entity. In those cases the entity used for the reference should be annotated
  - Example: "*The patient took **ADHD** prescription stimulants*"
    - **ADHD** should be annotated as a SYMPTOM
    - "**ADHD** prescription stimulants" should not be annotated as there is no drug in the list that could be found by looking for that concept.
  - Example: "*The patient received **fatigue** treatment*"
    - "***fatigue***" should be annotated as a symptom.
    - "***fatigue** treatment*" should not be annotated as there is no drug in the list that could be found by looking for that concept.

Attributes

- When an entity cannot be found in the list of concepts, "-1" will be used as the corresponding Entity Identifier.
  - All the annotations should have a value for the attribute Entity Identifier.
    - The -1 value can not be use for DRUGS (All annotated DRUGS have to be in Table A1).

Relations

- It is allowed to annotate relations between entities even if the related entities are not in the same sentence.
  - Example: "*The patient took **Adderall** during the day. As a result the patient's **concentration** improved*".
    - The entities to be annotated are **Adderall** (DRUG), and **concentration** (SYMPTOM). There will be a relation "**outcome-positive**" between these two entities even if each entity belong to a different sentence.

What _NOT_ to annotate?

Entities

- Entities should not both start and end with parenthesis.
  - In case this happens only the entity within the parenthesis will be annotated.
- DRUGS that are not listed in the Table A1 should not be annotated.
- In our annotations we don't allow co-reference nor anaphoric references.
  - Example: "***Geodon** used to make me **sleep**...now with **Adderall** and **Ritalin** at night? Nope*"
    - In that tweet "*Nope*" could be understood as "*No **sleep***", but we don't annotate that concept because we don't annotate anaphoric mentions.
  - Example: "Respondents used stimulants mostly for wakefulness and performance enhancement"
    - **In this example "stimulants" is not listed among our drugs, so it should not be annotated**
  - Example: "*I took **Geodon** yesterday. It doesn't work anymore*"
      - In this sentence "*It*" could be understood as "***Geodon***", but as we don't allow

anaphora "*It*" will not be annotated.

- **EXCEPTION:** When an entity that has to be annotated contains an anaphoric mention to another entity to be annotated, the entity containing the anaphora should be annotated using that context information.
  - In the sentence "*the patient experienced* ***Severe imbecility****, and that* ***imbecility*** *was intensified with the presence of […]*" the second occurrence of "**imbecility**" refers to "**Severe imbecility**", and should be annotated as such (**Severe imbecility**, with ID=10040442).

Attributes

- If the DRUG is negated the relation will not be annotated.
  - Example: "*I did not take* ***prozac*** *and now I don't have a* ***headache***"
    - The relation between "**prozac**" and "**headache**" should NOT to be annotated.
  - If it is just the SYMPTOM/DISEASE what is negated we annotate the relation.
    - Example: "*I took* ***prozac*** *and now I don't have a* ***headache***"
      - The relation between "**prozac**" and "**headache**" has to be annotated.
- The attributes of the entities should not be included in the annotation span unless required by the tokenisation, or in case the entity is a concept per se.
    - Example: "***nondiabetics***" (annotate the entire word)
    - Example: "*no* ***pain***" (annotate only "**pain**")
    - Example: "***probable chronic fatigue syndrome***" (only annotate "**chronic fatigue syndrome**").
    - Example: "***Severe dengue***" (annotate the 2 words as "**severe dengue**" is a concept recognized by MEDDRA)
- Determiners and quantifiers are never included in concept annotation unless that represents a different concept.
  - Example: "*I took* ***prozac*** *and* ***adderall*** *and now I'm very* ***tired***", the DISEASE is "**tired**", not "very **tired**".
    - In this case "*very*" will be encoded using the attribute "*Severity*", setting it to "*severe*"
  - Example: "*The patient has* ***Severe imbecility***"
    - In this case "***Imbecility***" is a concept (ID=10021409), but "**Severe imbecility**" is a concept too (ID=10040442), so we would annotate "**Severe imbecility**"
      - In this case too "*Severity*" attribute will be "severe".

Appendix

Drug Name	Brand name(s)
Lisinopril	Zestril, Zestroretic, Prinzide, Prinivil, Tensopril
Prednisone	
Montelukast	Singulair, Pluralair, Montecarolo-10, Montecarloflo, Lovetas
Triamcinolone acetonide	Kenalog, Volon A
Topiramate	Topamax
Destroamphetamine sulphate	Adderall
Cortisone	Cortisone
Venlafaxine	Effexor, Trevilor
Buprenorphine	Suboxone, Cizdol, Subutex, Zubsolv, Bunavail, Temgesic, Buprenex, Norspan, Butrans
Sertraline	Zoloft, Lustral
Dextroamphetamine sulphate	Adderall
Methylphenidate hydrochloride	Ritalin, Concerta, Methylin, Medikinet, Equasym, Daytrana, Phenida, Attenta, Hynidate, Focalin
Modafinil	Modafinil, Alertec, Modavigil, Provigil
Citalopram	Citalopram, celexa, cipramil
Paroxetine	Paroxetine, paxil
Fluoxetine	Fluoxetine, prozac
Fluvoxamine maleate	Faverin, Fevarin, Floxyfral, Luvox
Carbamazepine	Tegretol
Olanzapine	Zyprexa, Zypadhera, Lanzek
Trazodone	Depyrel, Desyrel, Mesyrel, Molipaxin, Oleptro, Trazodil, Trazorel, Trialodine, Trittico
Ziprasidone	Geodon, Zeldox, Zipwell
Ciprofloxacin	Ciprofloxacin
Levofloxacin	Levaquin, Tavanic
Moxifloxacin hydrochloride	Avelox, Avalox, Avelon, Vigamox, Moxeza
Quetiapine	Seroquel
Bevacizumab	Avastin
Melphalan	Alkeran, Sarcolysin
Rupatadine	Rupafin, Alergoliber, Rinialer, Pafinur, Rupax, Ralif
Tamoxifen	Nolvadex, Istubal, Valodex, Genox
Docetaxel	Taxotere
Seroquel	Quetiapine
Lamotrigine	Lamictal
Duloxetine	Cymbalta
Lisdexamfetamine	Vyvanse, Venvanse, Elvanse, Tyvense

Table A1. Drug names and brand names of the targeted DRUGS.
